# Family medicine rotation in Botswana: experiences of fifth-year medical students in decentralized rural training sites

**DOI:** 10.11604/pamj.2023.44.67.38504

**Published:** 2023-02-02

**Authors:** Deogratias Ongona Mbuka, Aloysius Gonzaga Mubuuke

**Affiliations:** 1 Department of Family Medicine, University of Botswana, Maun, Botswana; 2 Department of Radiology, School of Medicine, Makerere University, Makerere, Uganda

**Keywords:** Decentralized training, rural training, family medicine rotation, fifth-year medical students’ experiences, medical student, Botswana

## Abstract

**Introduction:**

globally, medical students have been exposed to decentralized training in rural settings. The experiences of these students regarding this kind of training have been reported in various settings. However, such students´ experiences have rarely been reported from sub-Saharan Africa. The purpose of this study was to explore fifth-year medical students´ experiences of a Family Medicine Rotation (FMR) at the University of Botswana and their recommendations for improvement.

**Methods:**

an exploratory qualitative study using a Focus Group Discussion (FGD) was done to collect data from the fifth-year medical students who underwent family medicine rotation at the University of Botswana. Participants´ responses were audio-recorded and later transcribed. Thematic analysis was used to analyze the data collected.

**Results:**

the overall experience of the medical students during FMR was positive. Negative experiences included issues with accommodation, logistic support onsite, inconsistency of learning activities between teaching sites, as well as limited supervision attributed to staff shortage. Emerging themes from the data included: diverse FMR rotations experiences, inconsistency of activities and different learnings between FMR training sites, challenges, and barriers to learning during FMR, enablers to learning during FMR, and recommendations for improvement.

**Conclusion:**

FMR was regarded as a positive experience by fifth years for medical students. However, improvement was needed especially with the inconsistency of learning activities between sites. Accommodation, logistic support, and recruitment of more staff were also needed for the improvement of medical students´ FMR experience.

## Introduction

Medical students´ rural training is considered a solution to urban-rural health services inequalities in promoting rural retention regardless of a weak positive correlation between undergraduate exposure and future rural practice [1]. The success of a rural rotation in enhancing teaching and learning depends on available communication technology, the longitudinal nature of the rotation, the focus of the school curriculum on primary care, the use of a decentralized training platform, and the ability to respond to students´ needs [2]. A one-year longitudinal training has been suggested as a solution to geographic disparities among health professionals [3]. There is agreement on the need of transforming medical education through the development of relevant skills and competencies required to respond to patient and community needs in the context that strengthens the health system [4-7]. To support this transformation, medical schools are critical actors for improvement in the shift of training away from the tertiary institution toward rural decentralized teaching platforms (DTPs). This promotes an understanding of the context and social accountability of students [8]. It has been also reported that medical students exposed to holistic care and continuity of care valued and supported an early exposure to family medicine away from tertiary teaching hospital [8-10]. Possibly from close to a decade of exposure to family medicine, final years (5^th^ years) medical students have expressed the need for early rural exposure during their training [9,11]. This is despite challenges with logistical and technological support, and accommodation issues requiring improvement for a successful program [11]. However medical students were excited about potential rural clinical exposure learning, with independent working opportunities under good supervision [12]. There are several factors including institutional and contextual ones do influence the success of a decentralized community training program [13] and these should be considered when planning an extension of clinical training to community settings. Previous literature has reported that Family Medicine Rotations (FMR) had a positive effect on knowledge, attitude, and some skills acquired by medical students in primary care [14], including a positive impact on family physicians, and patients [15]. Research in Botswana on medical students, either explored the effects of rural exposure on students´ future choice of practice location [16] or explored fifth-year medical students´ experience of entire rural exposure during their medical training [17]. The specific living and learning experience of fifth-year students on FMR has not yet been reported in Botswana. Considering students´ request of introducing family medicine in the curriculum as the first rotation [9], and based on final year medical students need for rural training [10], in addition to the unique context offered by decentralized sites for learning [13], the University of Botswana (UB), Department of Family Medicine and Public Health (DFM&PH) started training with decentralized sites away from the central campus. The early rural exposure of 32 weeks spread over the years of study was adopted. Sixteen weeks were spent under public health during their first, second, and fourth years of training. The remaining 16 weeks were spent in FMR, in their third and fifth year for eight weeks every time they were attached [16,17]. Sites living experiences and learning were expected to happen through undifferentiated patients´ biopsychosocial approach. Knowing students´ experiences of FMR in Botswana would be helpful for future training adjustments. Therefore, this study intended to explore fifth-year medical students´ experiences of FMR, enablers and barriers to learning, for improvement.

## Methods

**Design**: an exploratory qualitative study using a focus FGD to collect data was adopted. Eleven students participated in the FGD. Interacting with students in a FGD was viewed as an appropriate data collection approach since students were allowed to express their views on FMR living and learning experiences and provide suggestions for improvement.

**Setting and study population**: Maun and Mahalapye training sites were involved in the study since they hosted third- and fifth-year students. These sites operate in district hospitals which have 260 beds (Maun) and 270 beds (Mahalapye). Internal medicine, paediatrics, obstetrics-gynaecology, surgery/orthopedics, and other services like dermatology, ophthalmology, psychiatry, dental clinic, Ear-Nose-Throat (ENT), including a specialized orthopaedic service in Mahalapye, and oncology in Maun, are offered. Medical students rotated equally through the two sites. They spent eight weeks on the FMR site whenever deployed. Onsite students were attached to “four main clinical services (medicine, surgery/orthopaedics, paediatrics, obstetrics/ gynaecology)” and went to the clinic for outpatient exposure. Students were supervised and assessed by faculty supported by the district hospital staff and residents in family medicine (postgraduate students in family medicine). Expected teaching and learning were to happen during tutorials, Bedside teaching (ward round and calls), observed mini clinical examination exercise (Mini-Cex), outpatient consultations, cases presentations (three stages assessment and management plan format), directly observed clinical procedural skills (DOCPS), and participation to different weekly problem-based learning (PBL) sessions. Typically, each site received five groups of mixed (third- and fifth- years) medical students for two months starting from August of a year to May of the following year. Fifth-year medical students constituted the study population. Half of the enrolled eligible 39 fifth-year students were expected to rotate in either Maun or Mahalapye site.

**Recruitment and sample size**: students were verbally informed throughout the year by the site´s coordinators about the upcoming study at the end of the 2016 academic year. The study was conducted in June 2016, after the last group of students completed the FMR. Two representatives of students from the two streams (Maun and Mahalapye) volunteered to facilitate the recruitment of participants. They were in touch with the main researcher (Maun site manager) who reminded them when needed, to contact and invite colleagues to participate in the study in Gaborone. Follow-up calls were done to assure that all fifth-year students were back in Gaborone and were reminded about the FGD. To reduce participants´ bias, none of the site managers attended the FGD. We purposefully selected students who rotated twice in the same site and then those who rotated once in the two different sites to allow for variability of experiences on the two sites. All fifth years were eligible and invited to participate in the study. Those who declined participation were excluded. Eleven candidates of different expected categories out of the 39 fifth years responded to the FGD in June 2016. One FGD was organized with present students.

**Data collection**: the research assistant (MPH holder) UB employee, experienced in the qualitative study and data collection process, never involved with these students, facilitated the FGD in Gaborone. The FGD lasted for about two hours. Open-ended questions were used with the objectives of exploring fifth years´ experience of FMR, enablers, and barriers to learning, and suggestions for improvement as variables of interest. Probing, clarification, and reflective summary were done, while approval or disapproval with appropriate correction of summarized interaction content by participants to FGD for transactional credibility [18] was done as member check. Field notes were used to complement the FGD transcript for triangulation. The discussion was done in English and the process was audiotaped followed by a verbatim transcription. Nicknamed participants as black (1), white (2), red (3), grey (4), yellow (5), blue (6), purple (7), brown (8), pink (9), orange (10) and green (11), were referred to in the result section as P1 to P11. Only clearly expressed quotes were used.

**Data analysis**: thematic analysis was employed. Researchers familiarized themselves with the transcripts, separately coded and categorized responses which were then organized into emerging themes, according to the framework approach [19]. Different codes, categories, and themes between researchers were harmonized for the final write-up. Atlas ti Version 7.5.18 was used to organize and capture the findings. Transferability of findings and dependability of the study were assured by setting and study processes description.

**Ethical considerations**: permission to conduct this study was granted by the MOH / UB Ethical Review Board (HPDME: 13/18/1 Vol. X (297). To assure confidentiality, participants´ details were not included in the report. Audio records and transcripts, notes were kept safe by the principal investigator in a safe lockable cabinet. Both researchers involved in the analysis of the data had access to them. Participants signed consents before the FGD.

## Results

**Participants characteristics**: eleven fifth-year medical students of median age 24(7) years, of which seven (63.6%) were female, participated. About half (n=6.54%,) of participants rotated once in the two training sites (Table 1).

**Table 1:** focus group discussion participants´ characteristics

Variables	Characteristics	Participant N (%), Median (IQR)
**Age**		24(7)
**Sex**	Male	4(36,4%)
	Female	7(63.6%)
**Participants and Number of rotations per site**	Mahalapye rotation twice	3(27.3%)
	Maun rotation twice	2(18.2%)
	Mixed rotation (One in Maun and one in Mahalapye)	6(54.5%)

**Emerging themes and categories**: diverse FMR experiences, inconsistency of activities and different learnings between FMR training sites, challenges, and barriers to learning during FMR, enablers to learning during FMR, and recommendations for improvement of FMR emerged (Table 2).

**Table 2:** emerging themes and categories from the focus group discussion (FGD) with fifth-year medical students

Emerging themes	Categories
Theme 1: Diverse FMR experiences	From Beneficial, useful, and overwhelming to relaxing or inadequate.
Theme 2: Inconsistency of activities and different learnings between FMR training sites	Different administrative standard between two training Sites; Different Supervision experiences between the two sites; Different topics and teaching methods with influence on student performance on assessment; Different learning between Mahalapye and Maun
Theme 3: challenges and barriers to learning during FMR	Insufficient onsite staff; Starting clinical rotation with family medicine; Accommodation, communication issues and poor home internet
Theme 4: Enablers to learning during FMR	Proximity to faculties and residents and being part of the teamwork; Mentorship and library internet access
Theme 5: Recommendations for improvement of FMR	Employment of more staff trained in family medicine; Joint third- and fifth-year group allocation on sites to allow close guidance and student interactions; Clear details schedule of site activities; More involvement of residents in teaching and supervision activities; Consistency in teaching activities supported by a commonly used document in the two sites; Closer to training site accommodation needed

### Theme 1: diverse experiences of FMR

**From beneficial, useful, and overwhelming to relaxing or inadequate**: the individual medical student had a unique experience with FMR. It was experienced as an opportunity for revision for some. Others considered it as a break from the tension at Princess Marina Hospital (PMH) in Gaborone, contrasting with the overwhelming weekly topics experienced by others during FMR. *“I was able to revise …that was the good thing about it” (P7); “We have three topics per week and one topic can take a week on itself. So, we felt overloaded, I mean three major topics per week, there is too much to cover(P11);” “You get two months away from Marina, away from Gaborone, it is just a break”*(P9).

Diverse experiences were summarised as: “Necessary” (P11); *“it was helpful”* (P10); “*amazing*” (P3); “*beneficial*”(P6); “*Crucial for skills*” (P4); “*Room for improvement*” (P1); “*It is good but could be better*” (P8); “*different from other rotation*” (P9); “*relaxing*” (P7); “*Lacking*” (P2); “*good but inadequate*” (P5).

### Theme 2: inconsistency of activities and learnings between sites

Medical students reported inconsistency in administrative issues, supervision, teaching, and overall learning between the two sites:

**Different administrative standards between the two training sites**: students felt that there was a double standard across a variety of administrative accountability issues, between the sites with an absence of uniformity in teaching activities resulting in a comparison of the two sites on good or bad doing: *“in Mahalapye it is not compulsory for students to come and spend a night in the hospital while in Maun it is. And it is not the only thing even in terms of how long you should be in the hospital, in Maun and Mahalapye it is completely different. When I speak to my colleague in Mahalapye they are missing days, and they are leaving early. “We are not doing the same thing” (P11)*.

**Different supervision experiences between the two sites**: there was either satisfaction or dissatisfaction or else an opposing opinion regarding the type of supervision received in a training site. Family physicians´ and residents ´ involvement were different in the two sites including the inconsistency of supervision within a given site when students compared their experiences during the third and fifth year of attachment in the same site with opposing opinion on the on-site quality of supervision.

*“What was surprising is that in Maun the specialist goes with you guys to the clinic that was in the third year when I was there, in Mahalapye you end up transporting yourself, they through you with residents*” (P1). “*Another issue that was good about Mahalapye because I was there that their residents are very helpful even if our course coordinator is not, they were able to do with us the other activities*” (P2); “*In Maun, they [residents] are difficult to find, you only find them when you are doing PBL and maybe when you are on call a few residents were available*” (P7).

“*Yes, so we had constant supervision when we were in Mahalapye; There was always somebody to guide us. In Maun once we departed from the clinic, we were in the ward with the doctor so there was very limited supervision*”(P5); “*I want to go back to what*(P5) *said earlier on the contact that they had with the supervisor in Mahalapye, that they had good contact with a supervisor in Mahalapye, in the third year she felt that she was learning a lot, but that was not always the case when I was doing my fifth year in Mahalapye*” (P8).

**Different topics and teaching methods with influence on student performance**: students noted a discrepancy in teaching methods and choice of topics with potential impact on their performance during assessment which would favor the group that was taught a certain skill and surprise those who were not taught. “*Another thing to point out is that in the third year I went to Maun while in the fifth year I went to Mahalapye; well, I appreciated a bit of discrepancy in two places, I mean how they do things how they do their clinic is different. Something worth being exposed to in Mahalapye may not have happened in Maun and may be unfair because you can get a question in an exam where somebody said we were taught this in Mahalapye while someone else says ‘Nya’ [no] we were not taught that in Maun*” (P1).

“*I think it will be beneficial if they teach us how we are supposed to do a blood pressure measurement, how to do a knee examination so that when we get to the exam, we do not get surprised”*(P7).

**Different learning between Mahalapye and Maun**: the group was hesitant to express their compared learning sites experiences, however, one of the students, expressed satisfaction with Maun training, while another suggested that Mahalapye was the best.

*“Ok I need to say that I was in Maun for family medicine rotation both third and fifth year, I enjoyed myself there*” (P11); “*Mahalapye, Mahalapye is the best”* (P1).

### Theme 3: challenges or barriers to learning during FMR

The number of onsite staff, starting family medicine as the first clinical rotation, and issues around accommodation, internet, and administrative communication were considered obstacles to learning in FMR.

**Insufficient onsite staff**: the inadequate number of Family physicians and other specialists was considered a barrier to the learning process. *“I think a very important point is the lack of lecturers. You will see that in the ward [like] internal medicine especially, the specialist only comes on Monday, the rest of the weekday you are doing round with interns and medical officers, and I feel that they cannot adequately impact some information*” (P 11).

**Starting clinical rotation with family medicine**: starting clinical rotation with family medicine as a third-year medical student was challenging due to the broad field that is family medicine.

*“[…] You have never done any clinical experience, and you get there you are thrown into everything, and you do not remember most of the thing and it is very difficult if you are doing it as the first one especially if you are a third year*”(P2).

**Accommodation, communication issues, and poor home internet**: distant accommodation from the training site and unclear communication channel with the owner of the house, electricity and water quality in Maun, and poor home internet were seen as challenging environments for learning during FMR.

*“In Mahalapye the place we were staying was just too far we had a problem with transport when you go there and even when you come back, it limits me in terms of if I want to do more and get more exposure, so I need to go back. The problem is that if we had a closer accommodation*” (P3). “*In Maun, it is the same thing, the place where we stayed was far away from the hospital […] it is very difficult to get transport at night and late in the evening*” (P 10). “*Any time we wanted to contact our landlord it did not happen, and we did not have the contact for the administrator*” (P6). “*We have a horrible water situation in Maun, the water is brown, and during our third year, we had to draw water from the hospital*” (P10). “*The Wi-Fi is in the house but most of the time it is very slow so in Maun we could not get to do our PBL in the room. We always did that in the hospital*” (P10).

### Theme 4: enablers of learning during FMR

**Proximity to faculty, and residents, and being part of the teamwork, mentorship, and library internet access**: while residents in Mahalapye were more available compared to those in Maun, working closely with faculty and residents enabled learning. The family medicine teamwork, the mentorship initiative, and library Wi-Fi internet access promoted learning.

*“Mahalapye residents were available while in Maun there were not always around*” (P7). “*Working closer to a university of Botswana (UB) person, resident, and being part of the teamwork*“(P5). “*I think the teamwork was appreciated [both sites], because in places like PMH the teamwork between the nurses, doctors, and students was not the same, in family medicine you do a lot of skills, you feel motivated someone doing cannula someone else doing something else and you feel encouraged*”, “*Mentorship program to help third years is a god enabler*” (P10).”*The access to Wi-Fi. Library was an enabler*” (P5).

### Theme 5: recommendations for improvement

For a better FMR experience, an individual medical student (P) and a group (G) recommended the following: 1) employment of more staff trained in family medicine: “*more staff, more family medicine specialists*” (G); 2) joint third- and fifth-year group allocation on sites to allow close guidance and student interactions.

*“I am saying that third years and fifth years should be always together, the third year should have fifth years, and the fifth year should have heard third years”*(P11); 3)more involvement of residents in teaching and supervision activities. “*The residents should be more involved*” (P1); 4) clear details schedule of site activities: “*like a daily schedule, if I am not having a lecture, what should I be doing at a given time*” (P11). 5) consistency in teaching activities supported by the commonly used document in the two sites: “*yes I was saying consistency, also we also need that, but that can also be covered when we have clear objectives that are documented and expanded and both Mahalapye and Maun have those documents they know what they should follow then it will be easier to have consistency*” (P2); 6) closer to training site accommodation needed: “*Accommodation closer to the hospital”*(G).

## Discussion

This study intended to explore fifth-year medical students´ experiences of FMR. Experiences varied among participants. For some, it was different from other rotations, helpful, necessary, beneficial, crucial for skills, amazing, relaxing, and contrasting with it being inadequate requiring improvement for other participants. These diverse experiences from good to frustrating were also reported in South African settings [12]. In a study on the rural experience of medical students conducted in Botswana two years before this one (2014), a lack of teaching during FMR was reported, implying that it was not beneficial [17]. This negative perception is to be considered in a wide range of experiences which predominantly was positive in this study. The onsite teaching has since been introduced in the form of chosen scheduled tutorials and times of activities shared with students at each beginning of FMR for the intended eight-week periods. The positive perception expressed of FMR in this study is not an isolated perception for Botswana. FMR was reported as a good or positive experience by medical students elsewhere [5,6,9-12]. It was also anticipatively considered as a positive experience by medical students based on presented expected training outcomes [12], hence should stand as an important component of the training of future doctors despite some negative experiences requiring improvement.

Perceived inconsistency on administrative stands, training activities as well as consequent different learning outcomes between the two training sites of FMR were also reported. Although not in terms of inconsistency between the two sites, rostering of supervisors within a training site was reported in South Africa [11]. This has been implemented by assigning a facilitator for activities as part of the eight weeks of scheduling. The consideration of the consensus key elements for decentralized training [2], is crucial in the development of new sites. The decentralized nature of the DFM in Botswana with two distant training sites away from the headquarter could be mainly responsible for these findings. Additionally, the discrepancy between sites in decentralized training is inherent to its nature since learning in any site depends on site dynamics, site leadership, and the type of interaction between students, lecturers, health professionals, and the community in a particular setting [13]. These interactions and the rural setting of FMR are crucial in the transformation of medical education with the potential for the production of graduates suitable for the setting and community needs [8], toward which the UB is aiming. However, efforts to minimize inconsistency between the two sites were done by providing a similar schedule of activities, harmonizing onsite tutorial topics, stands on administrative arising issues, and having regular exchanges between site managers on teaching and topics to be considered. Despite the overall positive perception of FMR by participants, they reported a range of challenges due to self, logistical support, and insufficient onsite staff, including issues related to the site environment and accommodation. Starting rotation with FMR was challenging compared to doing it following other clinical rotations especially when students were in their third year of training. Poor homes Wi-Fi connectivity, including the observed insufficient onsite staff member stretched and providing limited supervision were part of rural training challenges. This limited supervision is similar to that reported in a previous study on rural experiences of medical students [17] who happened to have spent half of their rural exposure in FMR. Inadequate staff for adequate supervision was also reported somewhere else during FMR [14]. Onsite staff number should therefore be increased.

Staff shortage has not yet been fully addressed, however alternative solution from students´ recommendations like more resident involvement in teaching and supervision has been implemented. Grouping third-and fifth-year students in interactive groups to guide the first steps of third-years, supplementing supervision deficit, and promoting onsite team build-up, peer interaction, and integration to district teamwork allowing to learn the roles of each professional actor [11] were also implemented. This development will, however, need quality evaluation in the future for a meaningful good experience resulting in the rural choice of practice outcome [12,17], while the staffing situation in hosting training sites should be considered seriously. Distant accommodation at the training site and poor quality of water were also challenging to learning. These were also experienced in terms of logistics, technology, and accommodation issues [11], needing attention for optimization of student learning experiences. These challenges are however part of the complex interactions where students are to be trained and which are sites specific factors [13], and responsible for either individual or group different experiences of FMR. Reliable onsite internet for better connectivity has been installed, and students have been relocated to accommodation near training sites.

Interaction with lecturers and residents was viewed as an enabler of learning. However, the implication of district hospital professionals in teaching should be encouraged to promote multidisciplinary learning expressed elsewhere during FMR [11]. The role of WIFI was also acknowledged by these authors as being an enabler of learning. The important role of recommended onsite scheduling and site supervisors´ assignment was also reported elsewhere [11] and considered crucial for the resolution of site inconsistency reported in this study. Finally, the distance between training sites and medical students´ residences was addressed since accommodation has bearing on training [11]. Limitations: study findings are not generalizable to another cohort of students in UB or other countries. Findings are specific to the period of the study as current perceptions may have evolved by ongoing changes to address the issue reported before this write-up. Self-identified participants may have either positive or negative biases to current findings. Lastly current findings may not be exhaustive as a second FGD would have allowed the identification of potential new themes if any, in terms of saturation [20].

## Conclusion

Final-year medical students reported a positive perception of their FMR. The decentralized nature of FMR training sites and the reported discrepancy between sites require attention to address the factors raised by the students. The recruitment of more staff per site will certainly contribute to a better FMR experience for students at UB.

### What is known about this topic

- *Rural training of medical students has the potential to promote a future choice of rural setting practice;*
- *Family medicine rural training of medical students is a skill learning, holistic approach training opportunity desirable to start early in the training;*
- *Decentralized training sites have specific sites interconnected dynamics that influence different students learning*.

### What this study adds

- *Medical student training in decentralized multicenter rural family medicine sites is prone to inconsistency in teaching and supervision within and between distant training sites;*
- *There is a need for manpower and synchronized teaching activities planning to minimize inconsistency between rural training sites for a good rural experience of family medicine exposure*.
